# Analysis of spino-pelvic sagittal parameters in patients with highly downward-migrated lumbar disc herniation

**DOI:** 10.3389/fsurg.2026.1746593

**Published:** 2026-05-20

**Authors:** Li Huang, Yue Li, Yanjie Wang, Qingqing Xiao, Siyu Kou

**Affiliations:** Neck-Shoulder and Lumbocrural Pain Devision 1, Sichuan Province Orthopedic Hospital, Chengdu, China

**Keywords:** highly downward-migrated lumbar disc herniation, pelvic morphology, pelvic retroversion, sagittal balance, spino-pelvic parameters

## Abstract

**Objective:**

To investigate the characteristics of spino-pelvic sagittal alignment in patients with highly downward-migrated lumbar disc herniation (HDM-LDH).

**Methods:**

A retrospective analysis was conducted on 149 hospitalized patients diagnosed with HDM-LDH, and 138 asymptomatic adults undergoing physical examination were enrolled as the control group. Sagittal alignment parameters including thoracic kyphosis (TK), thoracolumbar kyphosis (TLK), lumbar lordosis (LL), sacral slope (SS), pelvic tilt (PT), pelvic incidence (PI), PI-LL, and sagittal vertical axis (SVA) were measured on standing full-spine lateral radiographs. The differences in these parameters between the two groups were analyzed.

**Results:**

Compared to the control group, the HDM-LDH group exhibited significantly lower LL and SS, whereas PT, SVA, and PI-LL were significantly higher (*P* < 0.05). No significant differences were found in PI, TLK, or TK between the groups. In spinal parameters, LL was moderately correlated with PT and SS, and weakly correlated with PI and TK. Regarding pelvic parameters, PI was moderately correlated with SS and PT, while PT showed a weak correlation with SS; all were statistically significant. SVA was weakly correlated with LL. The predominant lumbar lordosis subtype in the HDM-LDH group was Roussouly type II (SS < 35°, reduced LL, apex at L4). The proportions of types I, II, and IV were higher in the HDM-LDH group, while type III was less prevalent compared to the control group (*P* < 0.05).

**Conclusion:**

Patients with HDM-LDH exhibit distinct spino-pelvic sagittal characteristics compared to asymptomatic individuals, characterized by loss of lumbar lordosis, pelvic retroversion, and a nearly straight spinal alignment with horizontally oriented intervertebral discs. These morphological features alter the biomechanical structure of the spine and may underlie the pathogenesis of downward-migrated disc herniation.

## Introduction

Lumbar disc herniation (LDH) is a common degenerative spinal disorder characterized by compression of the dural sac or nerve roots within the spinal canal, primarily resulting from intervertebral disc degeneration and various contributing factors, which ultimately lead to a range of clinical symptoms (1). Choi et al. assessed the extent of herniated nucleus pulposus migration on T2-weighted sagittal MRI images, using the height of the posterior margin of the intervertebral disc as a reference. Migration exceeding this height was defined as high-grade. Highly downward-migrated lumbar disc herniation (HDM-LDH) specifically refers to cases in which the nucleus pulposus migrates inferiorly beyond the posterior margin of the affected disc level. These free fragments are typically located between the medial aspect of the pedicle and the pars interarticularis of the lumbar vertebrae (2). However, the pathogenesis of HDM-LDH remains poorly understood.

Recent studies have demonstrated that spino-pelvic sagittal alignment plays a crucial role in the development of degenerative lumbar conditions, with pelvic incidence (PI) recognized as a central morphological parameter (3–7). To date, limited research has examined the characteristics of sagittal spino-pelvic alignment in patients with HDM-LDH. Therefore, this retrospective study aimed to investigate the sagittal spino-pelvic sequence in individuals with HDM-LDH, with the goal of enhancing our understanding of its underlying pathomechanism.

## Material and methods

### General information

This study was approved by the Medical Science Research Ethics Committee of Sichuan Provincial Orthopedic Hospital (Approval No. KY2024-008-01). Patients who were initially diagnosed with HDM-LDH between January 2020 and June 2024 were retrospectively selected via the electronic medical record system. The inclusion criteria were: (1) Diagnosis of HDM-LDH confirmed by clinical evaluation and imaging examinations; (2) Availability of complete standing full-spine anteroposterior and lateral radiographs, as well as lumbar CT images performed at our hospital. Exclusion criteria were: (1) Presence of other spinal conditions such as idiopathic scoliosis, spinal deformity, or lumbar spondylolisthesis; (2) History of spinal fracture, trauma, or tumor; (3) History of open or minimally invasive spinal surgery; (4) Incomplete examinations or unavailable imaging data.

Asymptomatic adults who underwent physical examination during the same period were included as the control group. The inclusion criteria for the control group were: (1) Age >16 years; (2) Absence of spinal disease confirmed by orthopedic evaluation; (3) No history of spinal, hip, or pelvic disorders; (4) No contraindications for x-ray imaging (e.g., pregnancy, tumor); (5) Complete standing full-spine anteroposterior and lateral radiographs available.

### Imaging indicators

MRI and CT were utilized for all patients to confirm the high-grade downward migration. All imaging was performed in the Department of Radiology at our hospital using weight-bearing (standing) full-spine lateral radiographs, including both femoral heads. The x-ray source was fixed, and images were automatically stitched following continuous exposure. To prevent lower back and leg pain from compromising the examination results, patients experiencing severe pain received analgesics prior to the imaging. Most patients did not require analgesics, and interventions were only administered to those experiencing severe low back and leg pain that might affect the imaging results. Specifically, patients with a VAS score ≥ 6 (10 in 149 patients) received an intramuscular injection of 100 mg of tramadol hydrochloride, while those with a VAS score < 6 (25 in 149 patients) received 50 mg of diclofenac sodium enteric-coated tablets orally. Furthermore, to avoid posture-induced errors, patients were instructed to maintain an upright stance, look straight ahead, and fully extend their knee and hip joints during the radiography. Additionally, the elbows were fully flexed with both fists placed on the ipsilateral clavicles to prevent the upper limbs from interfering with the spinal imaging (8). Radiological analysis was conducted using the hospital's Picture Archiving and Communication System (PACS). All sagittal plane parameters were measured twice for both groups of patients by the same researcher under double-blind conditions with an interval of 2 weeks to assess intraobserver reliability. The angle measurement tool plugin within the system was accurate to 0.01°, and the distance measurement tool plugin was accurate to 0.01 mm. The average of the two measurements was calculated and recorded for the final statistical analysis.

**Figure 1:**
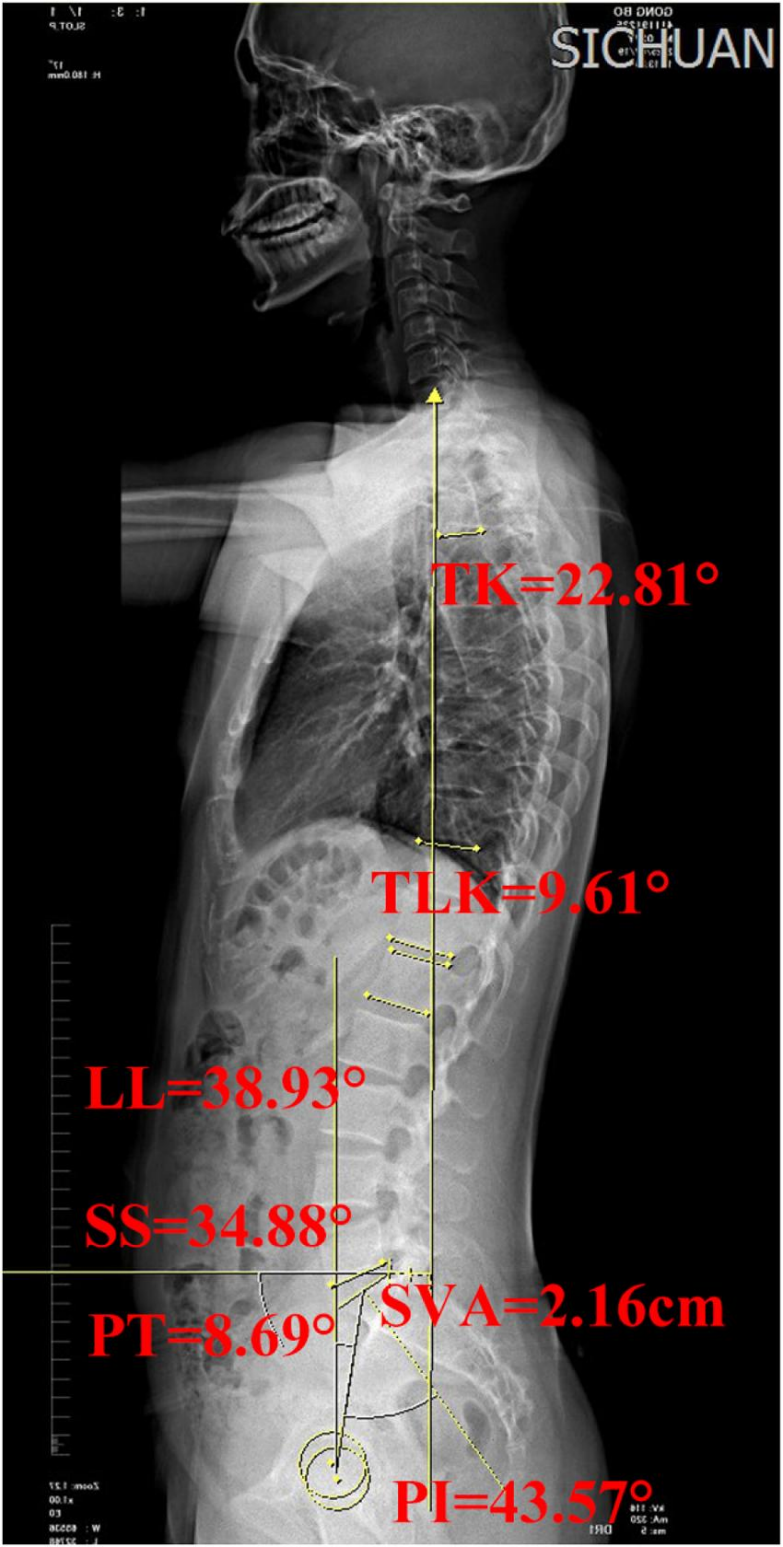
Schematic diagrams illustrating the spinal-pelvic parameters of an adult without HDM-LDH. PI, pelvic incidence; SS, sacral slope; LL, lumbar lordosis; TK, thoracic kyphosis; PT, pelvic tilt; SVA, sagittal vertical axis; TLK, thoracolumbar kyphosis.

The following key spino-pelvic sagittal parameters were measured on lateral radiographs (Figure 1): Thoracic kyphosis (TK): the angle between the superior endplate of T4 and the inferior endplate of T12 (9); Thoracolumbar kyphosis (TLK): the angle between the superior endplate of T11 and the inferior endplate of L1; Lumbar lordosis (LL): the angle between the superior endplate of L1 and the inferior endplate of L5; Sacral slope (SS): the angle between the sacral endplate and the horizontal line; Pelvic tilt (PT): the angle between the vertical line and the line connecting the midpoint of the sacral endplate and the midpoint of the femoral head line; PI: the angle between the line perpendicular to the sacral endplate and the line connecting the midpoint of the sacral endplate with the midpoint of the femoral head line; Sagittal vertical axis (SVA): the horizontal distance between the C7 plumb line and the posterosuperior corner of the S1 vertebral body (10).

Based on the classification proposed by Roussouly et al., lumbar lordosis was categorized into four types: Type I: SS < 35°, small LL, apex of lordosis at the midpoint of L5, nearly horizontal lumbar curvature; Type II: SS < 35°, small LL, apex at L4, horizontally oriented lower lumbar curve, with reduced thoracic and lumbar curvatures; Type III: SS between 35° and 45°, relatively large PI, apex at L4, typically involving four vertebral segments; Type IV: SS > 45°, apex at L3 or above, pronounced thoracic curvature (11).

### Statistical analysis

Statistical analysis was performed using SPSS version 23.0. Continuous variables conforming to a normal distribution were expressed as mean ± standard deviation (*x* ± *s*), and group comparisons were conducted using independent sample *t*-tests. Non-normally distributed data or data lacking variance homogeneity were analyzed using the Mann–Whitney *U* test. Categorical data were presented as counts and percentages, with group comparisons analyzed using the chi-square (*χ*^2^) test. A significance level (*α*) of 0.05 was applied. A *post-hoc* power analysis was conducted based on the core sagittal parameters (LL and PI-LL). With the current sample sizes (149 in the HDM-LDH group and 138 in the Control group), the study achieved a statistical power of >99% to detect the observed differences for both parameters, even after adjusting for multiple comparisons. Pearson correlation coefficients were used to assess relationships between parameters: *r* > 0.7 indicated a strong correlation; *r* = 0.4–0.7 indicated a moderate correlation; *r* = 0.2–0.4 indicated a weak correlation; *r* < 0.2 indicated no correlation (12).

## Results

### General information

**Figure 2:**
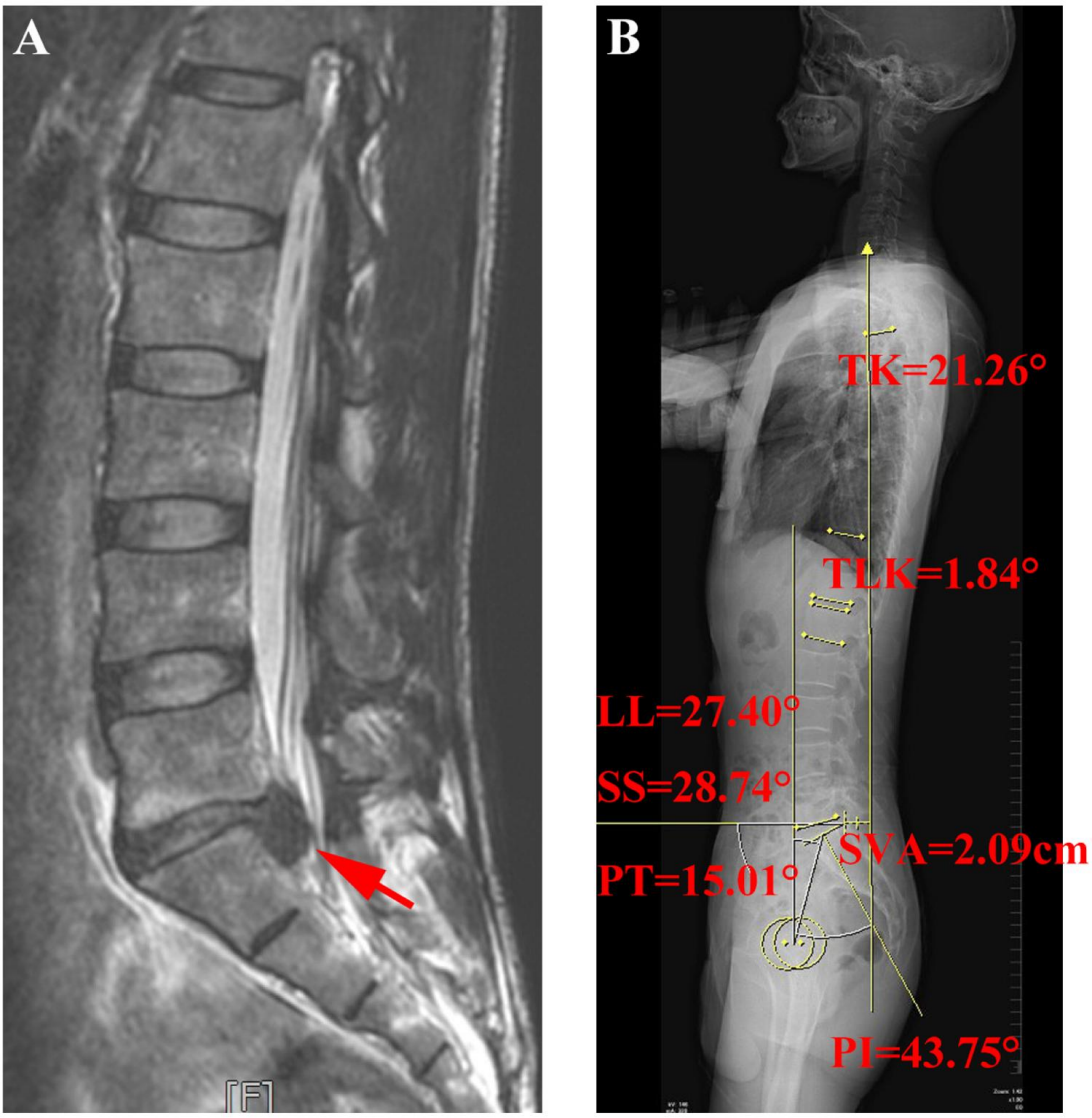
MRI imaging and spinal-pelvic parameters of a patient diagnosed with HDM-LDH. **(A)** Sagittal MRI. **(B)** Measured spinal-pelvic parameters. White arrows indicate the location of disc herniation. Patient information: male, 37 years old; diagnosed with L5–S1 disc herniation; preoperative MRI reveals a high-grade downward migration of the herniated fragment at the L5–S1 level. PI, pelvic incidence; SS, sacral slope; LL, lumbar lordosis; TK, thoracic kyphosis; PT, pelvic tilt; SVA, sagittal vertical axis; TLK, thoracolumbar kyphosis.

A total of 149 patients with HDM-LDH were included in the study group, comprising 92 males and 57 females, with a mean age of 50.28 ± 11.91 years. The distribution of herniation levels was as follows: L3/4 in 6 cases (4.0%), L4/5 in 87 cases (58.4%), and L5/S1 in 56 cases (37.6%). During the same period, 138 asymptomatic adults without lumbar or leg pain who underwent physical examination at our hospital were included as the control group. This group consisted of 88 males and 50 females, with a mean age of 31.73 ± 11.10 years. A case of HDM-LDH patient was shown in Figure 2.

### Comparison of spinal-pelvic sagittal plane parameters between the two groups

Compared to the control group, the HDM-LDH group exhibited significantly lower LL and SS, while PT, SVA, and PI-LL were significantly higher (*P* < 0.05). No statistically significant differences were found in PI, TLK, or TK between the two groups (see Table 1).

**Table 1:** Comparison of sagittal spinal-pelvic parameters between control and HDM-LDH groups.

Variable	HDM-LDH	Control	*t* value	*P* value
	*n* = 49	*n* = 138		
PI (°)	46.39 ± 8.30	46.08 ± 5.77	0.360	0.719
PT (°)	15.55 ± 7.38	13.42 ± 5.47	2.751	0.006
SS (°)	30.84 ± 7.16	32.64 ± 5.17	−2.431	0.016
LL (°)	29.20 ± 9.89	37.68 ± 5.58	−8.846	<0.001
TLK (°)	6.69 ± 4.57	5.86 ± 3.59	1.709	0.088
TK (°)	23.75 ± 8.24	24.97 ± 4.06	−1.567	0.118
SVA (cm)	3.91 ± 3.21	2.76 ± 1.31	3.935	<0.001
PI-LL (°)	17.58 ± 10.42	8.69 ± 5.17	9.039	<0.001

PI, pelvic incidence; PT, pelvic tilt; SS, sacral slope; LL, lumbar lordosis; TLK, thoracolumbar kyphosis; TK, thoracic kyphosis; SVA, sagittal vertical axis; PI-LL, pelvic incidence minus lumbar lordosis.

### Comparison of lumbar lordosis types between the two groups

In the HDM-LDH group, Roussouly type II was the predominant subtype of lumbar lordosis. The proportions of patients classified as Roussouly types I, II, and IV were higher in the HDM-LDH group than in the control group, while the proportion of Roussouly type III was lower. These differences were statistically significant (*P* < 0.05; see Table 2).

**Table 2:** Comparison of different Roussouly types between control and HDM-LDH groups.

Group	Type Ⅰ	Type Ⅱ	Type Ⅲ	Type Ⅳ
HDM-LDH	19 (12.8%)	93 (62.4%)	31 (20.8%)	6 (4.0%)
Control	0 (0%)	78 (56.5%)	60 (43.5%)	0 (0%)
*χ* ^2^	35.188			
*P*	<0.001			

### Correlation of spinal-pelvic sagittal plane parameters

Significant correlations were observed between spinal and pelvic parameters. For spinal parameters, LL showed moderate correlations with PT and SS, and weak correlations with PI and TK. For pelvic parameters, PI showed moderate correlations with SS and PT, while PT had a weak correlation with SS, all of which were statistically significant. Regarding global sagittal balance, SVA was weakly correlated with LL (see Table 3).

**Table 3:** Correlation of sagittal spine-pelvis parameters refer to the HDM-LDH group.

Variable	PI	PT	SS	LL	TLK	TK	SVA
PI		0.580**	0.531**	0.284**	−0.179*	−0.028	−0.110
PT			−0.379**	−0.233*	−0.068	−0.131*	0.104
SS				0.562**	−0.132*	0.101	0.011
LL					−0.079	0.370**	−0.305**
TLK						0.179**	−0.075
TK							−0.119*
SVA							

PI, pelvic incidence; PT, pelvic tilt; SS, sacral slope; LL, lumbar lordosis; TLK, thoracolumbar kyphosis; TK, thoracic kyphosis; SVA, sagittal vertical axis; PI-LL, pelvic incidence minus lumbar lordosis.

* *P* < 0.05.

** *P* < 0.01.

## Discussion

Free sequestrated lumbar disc herniation accounts for a considerable proportion of LDH cases, with highly migrated types more often occurring distally than proximally (13, 14). This distribution can be explained by the anatomical features of the region. The posterior longitudinal ligament serves as a key anatomical barrier preventing disc migration (15). Additionally, other structures such as the midline septum and the epidural membrane restrict lateral movement of the nucleus pulposus, thereby making upward or downward migration more likely (16). These directions of migration often lead to compression of the dural sac, increasing the risk of neurological complications such as cauda equina syndrome. As the water content within the intervertebral disc diminishes, both disc tension and thickness decrease, leading to a narrowed disc space and reduced elasticity. Under sudden axial pressure, the disc may extrude and separate from its origin, eventually becoming sequestered within the spinal canal. These changes are particularly pronounced in areas lacking posterior longitudinal ligament coverage (17). Our findings suggest that altered spinopelvic alignment alters the biomechanical environment of intervertebral discs, which may contribute to the pathogenesis of HDM-LDH. However, it is important to note that the observed sagittal parameter characteristics are not unique to this condition. Similar alterations in sagittal alignment have been widely documented in other types of lumbar disc herniation and various degenerative spinal diseases (18–20). Therefore, rather than altered spinopelvic alignment acting as the sole driver, local anatomical structures, specifically the posterior longitudinal ligament, may be more directly involved in the pathogenesis and specific migration patterns characteristic of HDM-LDH.

Spinal degeneration is closely linked to the biomechanical configuration of the spino-pelvic complex, with sagittal alignment emerging as a critical factor in recent research (21, 22). Abnormal sagittal spino-pelvic alignment not only exacerbates degeneration but is also associated with spinal deformities, spondylolisthesis, and canal stenosis (23–25). Moreover, sagittal plane alignment is more closely related to patient pain and quality of life than coronal alignment (26). During human development, PI, PT, LL increase to accommodate changes in body weight and center of gravity. Once skeletal maturity is reached, PI remains constant, while PT and SS continue to adapt to postural demands (27). PI defines the anatomical relationship between the sacrum and femoral heads and determines the pelvis's compensatory potential (28, 29). In the present study, PI did not differ significantly between HDM-LDH patients and controls, indicating that the anatomical position of the sacrum relative to the femoral heads may not be the primary driver of HDM-LDH pathogenesis. PT and SS, as spatial orientation parameters, are subject to postural influence and compensate to maintain sagittal balance (11, 30). The geometric relationship PI = PT + SS implies that an increase in PT generally corresponds to a decrease in SS. Through pelvic retroversion, reflected in increased PT, the body adjusts the center of gravity to preserve upright balance. Compared with controls, patients with HDM-LDH exhibited significantly lower LL and SS and higher PT, SVA, and PI-LL. These findings suggest compensatory pelvic retroversion in response to altered spinopelvic alignment caused by loss of lordosis and anterior shift of the trunk. The spinal column in these patients presents a straighter profile, with horizontally aligned discs experiencing increased axial stress. This mechanical configuration likely facilitates annular rupture at the lower endplate, with the posterior longitudinal ligament guiding the herniated nucleus pulposus downward. During the compensatory process of pelvic retroversion, the excessive contraction of the lumbar extensor muscles not only affects biomechanical morphology, but the increased muscular force may also lead to elevated local lumbar pressure, which in turn promotes disc herniation and downward migration. Because PI is a constant morphological parameter, the lack of significant difference in PI between groups, combined with the observed differences in dynamic parameters like LL, strongly suggests that the altered sagittal alignment is likely a compensatory consequence of the lumbar disc herniation, rather than the initiating cause. The relationship between sagittal parameters and lumbar disc herniation is complex and potentially bidirectional.

Clinically, PI-LL is often used to assess spinopelvic congruity and surgical outcomes (31). A mismatch exceeding 20° is indicative of poor alignment and is frequently associated with worse pain and functional impairment (32). In this study, nearly half of the HDM-LDH patients exhibited PI-LL > 20°, suggesting impaired biomechanical harmony and a higher risk of progression. Spino-pelvic sagittal alignment is jointly influenced by spinal and pelvic parameters. To maintain visual and postural balance, the body may compensate through increased thoracic kyphosis, lumbar flattening, pelvic retroversion, or even hip and knee flexion (33, 34). In this study, LL was moderately correlated with PT and SS, and weakly correlated with PI and TK. PI showed moderate correlations with SS and PT, while PT was weakly correlated with SS. SVA was weakly correlated with LL. These correlations are consistent with theoretical biomechanical models. Furthermore, it is important to consider the effects of clinical variables on these measurements. Severe lower back and leg pain often forces patients to adopt a compensatory “painful posture”, which directly affects static sagittal x-ray measurements. The administration of analgesics to a small subset of patients experiencing severe pain prior to the examination was strictly intended to alleviate muscle spasms and postural distortion. This process eliminates pain-induced interference, ensuring that the spine reflects its true weight-bearing alignment and allowing for the acquisition of accurate and reliable data.

According to the Roussouly classification, lumbar sagittal morphology in HDM-LDH patients was most commonly type II (11). The majority of patients demonstrated small SS (<35°) and long, shallow LL. Their spinal alignment approached a linear configuration, with horizontally oriented discs subjected to concentrated axial loads. The high prevalence of L4/5 and L5/S1 herniations in our study underscores the vital interplay between specific herniation levels and global sagittal parameters. Notably, different Roussouly morphotypes alter the distribution of biomechanical loads across the spine, which may explain the localized vulnerability of these lower segments in our patient group. The unique morphological pattern may contribute to early disc degeneration and may be a predisposing factor for the development of HDM-LDH.

Several limitations of this study should be noted. First, the retrospective case-control design inherently limits causal inference. Therefore, our study cannot determine the initiating factor in the bidirectional relationship between sagittal parameters and lumbar disc herniation. Second, there is a substantial age difference between the HDM-LDH and control groups, and we acknowledge the missing demographic data regarding height, weight (or BMI), and chronic medical histories in the control group. Because age is a significant confounding factor that can affect sagittal parameters, readers are cautioned to interpret the parameter analyses in light of these baseline disparities. Future prospective studies utilizing strictly age-matched controls are necessary. Third, a notable limitation is the absence of a control group consisting of non-HDM-LDH patients (patients with regular, non-migrated lumbar disc herniation). Because of this, we cannot definitively rule out overlapping sagittal characteristics with other degenerative conditions. Future comparative studies including non-HDM-LDH patients are necessary to better isolate the specific biomechanical and anatomical variables that uniquely contribute to the development of HDM-LDH. Lastly, only SVA was included as a global sagittal parameter. Due to the limitations of our early imaging evaluation protocols, global angular parameters such as TPA and SSA were not captured, making the assessment incomplete in this dimension.

## Conclusion

Patients with HDM-LDH exhibit altered sagittal spino-pelvic alignment, including reduced LL and SS, and increased PT, reflecting compensatory posterior pelvic rotation. These morphological changes contribute to a straightened sagittal spinal profile and horizontally oriented intervertebral discs, which result in concentrated loading at the lower endplate and promote annular rupture. The anatomical constraints of the posterior longitudinal ligament may further facilitate downward migration of the nucleus pulposus. This study suggests that altered spino-pelvic sagittal alignment may alter the biomechanical environment of the intervertebral disc and contribute to the pathogenesis of HDM-LDH. However, these observed sagittal characteristics are not unique to HDM-LDH, suggesting that the altered alignment is likely a complex, bidirectional consequence of the herniation rather than the sole initiating cause. Additionally, excessive contraction of the lumbar extensor muscles during compensatory pelvic retroversion may further elevate local lumbar pressure and promote disc herniation. Future prospective studies, including non-HDM-LDH control groups, are necessary to definitively clarify this causal relationship.

## Data Availability

The original contributions presented in the study are included in the article/Supplementary Material, further inquiries can be directed to the corresponding author.
